# Application of Metabolomics in Traditional Chinese Medicine Differentiation of Deficiency and Excess Syndromes in Patients with Diabetes Mellitus

**DOI:** 10.1155/2012/968083

**Published:** 2012-06-13

**Authors:** Tao Wu, Ming Yang, Hua-Feng Wei, Song-Hua He, Shun-Chun Wang, Guang Ji

**Affiliations:** ^1^Center of Chinese Medicine Therapy and Systems Biology, Shanghai University of Traditional Chinese Medicine, Shanghai 201203, China; ^2^Institute of Digestive Disease, Longhua Hospital, Shanghai University of Traditional Chinese Medicine, Shanghai 200032, China; ^3^Department of Medicament, Longhua Hospital, Shanghai University of Traditional Chinese Medicine, Shanghai 200032, China; ^4^Department of Internal Medicine, Longhua Hospital, Shanghai University of Traditional Chinese Medicine, Shanghai 200032, China; ^5^Institute of Chinese Materia Medica, Shanghai University of Traditional Chinese Medicine, Shanghai 201203, China

## Abstract

Metabolic profiling is widely used as a probe in diagnosing diseases. In this study, the metabolic profiling of urinary carbohydrates was investigated using gas chromatography/mass spectrometry (GC/MS) and multivariate statistical analysis. The kernel-based orthogonal projections to latent structures (K-OPLS) model were established and validated to distinguish between subjects with and without diabetes mellitus (DM). The model was combined with subwindow permutation analysis (SPA) in order to extract novel biomarker information. Furthermore, the K-OPLS model visually represented the alterations in urinary carbohydrate profiles of excess and deficiency syndromes in patients with diabetes. The combination of GC/MS and K-OPLS/SPA analysis allowed the urinary carbohydrate metabolic characterization of DM patients with different traditional Chinese medicine (TCM) syndromes, including biomarkers different from non-DM patients. The method presented in this study might be a complement or an alternative to TCM syndrome research.

## 1. Introduction

Diabetes mellitus (DM) is a complex metabolic disorder characterized by chronic hyperglycemia, hypoinsulinemia, and ketosis. In 2000, around 171 million people were affected with DM. By 2030, this number is estimated to increase to 366 million [1]. Current statistics shows that over 10% of the world's aged population (60 years and above) suffers from this disease, and 90% of these patients have type 2 diabetes mellitus (T_2_DM) [2]. Diabetes always causes high morbidity and mortality rates due to chronic microvascular complications (e.g., retinopathy, nephropathy, or neuropathy) and macrovascular complications (e.g., ischemic cardiac problems, cerebral vascular accidents, and peripheral vascular disorders) [3].

In ancient China, DM was recognized as *xiaokezheng*, a disease with symptomatic polydipsia. Traditional Chinese medicine (TCM) has a long history of treatments for *xiaokezheng* [4]. According to TCM theory, Yin (things associated with the physical form of an object), Yang (things associated with energetic qualities), Qi (life force that animates the forms of the world), and Xue (dense form of body fluids that have been acted upon and energized by Qi) [5] are in an unbalanced state when people are suffering from a disease. Similarly, patients with DM could be classified as having deficiency syndrome or excess syndrome, which refers to the organs' insufficiency or excess in Qi, Xue, Yin, and Yang.

Metabolic profiling is defined as the quantitative measurement of the dynamic multiparametric response of a living system to pathophysiological stimuli or genetic modification [6]. The objective of metabolomics is to gain new insight into the pathophysiology of a disease and identify individual metabolites or profiles of metabolites as potential biomarkers that can distinguish between normal and pathological states [7]. Metabolomics has been used in the diagnosis and evaluation of diabetic patients [8] because of its effectiveness in evaluating systemic responses to any subtle metabolic perturbation. In addition, it has also been used in the identification of potential biomarkers [9].

Recent animal and human metabolomic studies have investigated the metabolic effects of oral glucose challenge [10–12], insulin resistance [13–18], type 1 [19, 20] or T_2_DM [20–28]. Previous studies investigated the metabolic profiling of plasma phospholipids in T_2_DM using liquid chromatography/mass chromatography (LC/MS) coupled with multivariate statistical analysis [29]. Methods based on plasma fatty acid profiles analyzed via GC/MS were also developed to investigate the differences between T_2_DM patients and healthy volunteers [30]. A multianalytical platform method using GC/MS and ultra performance liquid chromatography-mass spectrometry (UPLC/MS) was developed to obtain the global metabolite profiles of DM in rat models [31]. An imbalance between carbohydrate and lipid metabolisms is involved in the etiology and pathophysiology of diabetes. Therefore, a metabolic analysis is necessary to visualize the alteration of globally circulating metabolites in a person suffering from diabetes. In the present study, a metabolic profiling was performed using GC/MS of urinary carbohydrates in subjects with and without DM.

Partial least square linear discriminant analysis (PLSLDA) is currently the common method used in supervised linear modeling in the field of metabolomics. However, the relationship between the disease and metabolic data displays nonlinear characteristics in some cases. Therefore, nonlinear modeling has been applied in metabolomics [32, 33]. Recently, the “kernel trick” has been efficient in dealing with nonlinear problems. Kernel-based orthogonal projections to latent structures (K-OPLS) [34, 35] can considerably improve the predictive performance in situations where a strong nonlinear relationship exists. Model population analysis (MPA) was developed based on the idea of statistically analyzing the outputs of Monte Carlo Sampling (MCS)-derived “population” of models. The MPA-based method is expected to provide some comprehensive insights into the data because it allows the statistical analysis of some interesting outputs of several models. One typical MPA-based method can be used to identify important variables by examining the distribution of prediction errors of all the submodels [36]. Subwindow permutation analysis (SPA) was used in the present study to reveal informative metabolites by incorporating the Monte Carlo technique and strictly implementing the idea of MPA [37, 38].

Several diabetes-related studies have been reported in recent years. However, the metabolic profiles involved in the pathological processes of diabetes are yet to be addressed. Thus, the identification of biomarkers is needed for the adequate screening and diagnosis of diabetes. Syndrome differentiation is an important element in TCM theories and is the basis for the treatments of all diseases, including DM. Therefore, the TCM syndromes of patients with DM are necessary to characterize. However, previous studies have not revealed the differences among the urinary carbohydrate metabolites in the TCM syndromes of these patients. In the present work, we conducted a comparative analysis of 366 subjects using GC/MS combined with K-OPLS/SPA analysis to (1) compare the urinary carbohydrate profiles of subjects with and without DM, (2) compare the relationship between urine carbohydrate levels and TCM syndromes in subjects with DM, and (3) determine the characteristics and differences in TCM syndrome distribution between excess and deficiency syndromes.

## 2. Materials and Methods

### 2.1. Chemicals

Carbohydrate standards (C_4_ sugar 1, inositol C, talose, mannose, inositol D, glucose, inositol A, arabinose, xylose, and C_4_ sugar 2) were purchased from Sigma (St. Louis, MO, USA). Acetonitrile (HPLC grade), methanol (HPLC grade), and methylimidazole were purchased from Fisher/Aldrich (NJ, USA). Sodium borohydride (NaBH_4_), dimethyl sulfoxide, trifluoracetic acid, acetic acid, acetic anhydride, and chloroform (analytical grade) were purchased from Sinopharm Chemical Reagent Co. Ltd. (Shanghai, China). Water was obtained from a Milli-Q ultra-pure water system (Millipore, Billerica, USA).

### 2.2. Clinical Research Design

DM patients from the Tianlin Community Health Service Center, Shanghai city of P.R. China August 2009 to May 2010 were prospectively included in the study. All 366 samples included 308 patients with DM (241 deficiency and 67 excess samples) and 58 patients without DM as the comparison group.

Patients were required to abstain from eating greasy and sweet food before the study to avoid an interference with the metabolism of the human body. Study protocol was approved by the Ethics Committee of the Hospital, and a written informed consent was obtained from each respondent. Each blood sample collected in a fasting condition was immediately centrifuged at 3000× g for 10 min, and the plasma was transferred into a clean tube. All urine samples collected in fasting condition and plasma samples were stored at −80°C until analysis. 

### 2.3. Inclusion and Syndrome Differentiation Criteria

Based on the criteria formulated by the World Health Organization in 1999, DM is characterized by a fasting plasma glucose (FPG) of ≥7.0 mmol/L, a postload plasma glucose (2h PG) of ≥11.1 mmol/L, or a history of oral hypoglycemic or insulin use, or both [39]. TCM syndromes, including deficiency and excess syndromes, were differentiated according to the guidelines [40]. The information gathered from inspection, auscultation, and inquiring was obtained on the day of admission. Manifestations and other diagnostic information were determined independently by three experienced physicians to ensure an objective evaluation. If the three were in accordance, the subject will be included in the study. Otherwise, he/she will be excluded.

### 2.4. Exclusion Criteria

Patients suffering from other serious diseases involving major organs or infective diseases were excluded from the study. Moreover, those who cannot or are not willing to complete the study or those who had psychiatric disorders or intellectual dysfunctions were also excluded.

### 2.5. Clinical and Laboratory Assessment

Clinical data including date of birth, height, weight, body mass index (BMI), waist and hip circumference, systolic blood pressure (SBP), and diastolic blood pressure (DBP) were determined by a senior physician. Obesity is characterized by a BMI of ≥25.0 kg/m^2^ according to the Asian guidelines [41]. Serum levels of alanine aminotransferase (ALT), FPG, glycated hemoglobin (HbA1c), triglycerides (TG), high-density lipoprotein cholesterol (HDL-C), very low-density lipoprotein cholesterol (VLDL-C) in fasting condition, and 2h PG were measured using an automatic biochemical analyzer (Hitachi7180, Tokyo, Japan).

### 2.6. Sample Preparation of Urine for GC/MS

A 200 *μ*L sample of urine from each group was blended with 20 *μ*L of ammonia and 1 *μ*L of 0.5 mol/L NaBH_4_/dimethyl sulfoxide (DMSO). Acetic acid (100 *μ*L) was added dropwise to reduce the abundance of NaBH_4_ after the reduction reaction (120 min at 40°C). Acetylation (10 min at 40°C) was performed after adding 200 *μ*L of 1 methylimidazole and 1 mL of acetic anhydride. Subsequently, 2 mL of water was mixed with the extracts for 10 min at 40°C, and the mixtures were extracted with 2 mL of chloroform. The samples were centrifuged (4000× g for 10 min), and the supernatant was discarded. The samples were washed with 5 mL of water to remove the chloroform layer. The remaining layer was added with 1 g of sodium sulfate and taken for GC/MS. Allose (20 *μ*L) was used as an internal standard to be added into each 200 *μ*L sample.

### 2.7. GC/MS Conditions

GC/MS was performed using a Finnigan gas chromatograph (ThermoFinnigan, USA) coupled with a mass spectrometer (TRACE DSQ). A TR-5ms capillary column (60 m × 0.25 mm × 0.25 *μ*m, Thermo) was used in the gas chromatographic system. The inlet temperature was 250°C. Column temperature was increased from an initial 140°C to 198°C (2°C per min for 4 min). It was then programmed from 198°C to 214°C (4°C per min), 214°C to 217°C (1°C per min for 4 min), and 217°C to 250°C (3°C per min for 5 min). Inlet temperature was maintained at 250°C. Helium was used as a carrier gas at a flow rate of 1.0 mL/min. The GC/MS was injected with 1 *μ*L aliquots. The mass spectrometer was operated in electron impact and full-scan monitoring modes (m/z 40–450) with 0.2 s/scan velocity. Source temperature, electron energy, and solvent delay were set at 250°C, 70 eV, and 10 min, respectively.

### 2.8. Data Analysis and Software

All data were processed by the Xcalibur software (ThermoFinnigan, USA), and the detected peaks were aligned using hand integral methods. The ion peak area for each detected peak was normalized by NIST 05 Standard mass spectral databases in the NIST MS search 2.0 (NIST, Gaithersburg, MD, USA) software. Semiquantitative concentrations of urinary monosaccharides were obtained through the ratio of the peak area to the standard. The K-OPLS package (available at http://kopls.sourceforge.net/download.shtml) and Statistic toolbox of the MATLAB (version 7.1, Mathwork Inc.) software were used in the statistical treatment of the data and application of various multivariate methods. Parts of the source codes used in implementing SPA in MATLAB were freely available at http://code.google.com/p/spa2010/downloads/list.

Data are shown as mean ± standard deviations (SD). In addition, significance was expressed through independent *t*-tests for continuous variables and Pearson Chi-square tests for categorical variables using the SPSS 17.0 software (SPSS, Chicago, Ill, USA). Fisher's exact tests were calculated when the expected frequencies were less than 5 in any cell. A *P* value of <0.05 was considered to indicate statistical significance. 

### 2.9. K-OPLS Models for Classification

Based on our previous work [42] and related literature [34, 43], the K-OPLS model was employed in the present study to build a classifier, with *σ* as the parameter for the Gaussian kernel function. The kernel matrix K was centered to model estimation. The K-OPLS algorithm modeled the kernel matrix K through a set of predictive and Y-orthogonal components. Thus, the predictive score matrix and the Y-orthogonal score vector were estimated. After the estimation step of each Y-orthogonal component, K was deflated using the Y-orthogonal variation, followed by a subsequent updating of the predictive score matrix and further estimation of Y-orthogonal components. The kernel function parameter (*σ*) and the number of Y-orthogonal components (Ao) of the K-OPLS model were optimized using 10-fold cross-validation. All the samples were randomly partitioned into 10 equally sized folds according to their categories. Subsequently, 10 iterations of calibration and validation were performed. As a result, onefold of the data was held out for validation, whereas the remaining nine folds were used for calibration. Details on the model are provided in the previous work.

### 2.10. Revealing Informative Metabolites through Statistical Assessment of Variable Importance

Previous studies [37, 44] indicated that the SPA method used for uncovering informative metabolites is constructed based on the prediction error distribution of the K-OPLS models, which are based on the subdatasets obtained through Monte Carlo sampling in both sample and variable space. 

In the equation DMEAN_*j*_ = MEAN_*j*,*B*_ − MEAN_*j*,*A*_, MEAN_*j*,*A*_ and MEAN_*j*,*B*_ denote the mean prediction errors calculated by the normal K-OPLS and the latter permuted K-OPLS models of the *j*th metabolite, respectively. If DMEAN_*j*_ > 0, the inclusion of the *j*th metabolite in the K-OPLS model may improve the predictive performance. This type of metabolite is deemed as a candidate of informative metabolites in the present study. By contrast, if DMEAN_*j*_ < 0, the inclusion of this metabolite into a model may most probably reduce the predictive performance. Therefore, this type of metabolite is considered uninformative/interfering.

With these preparations, the informative metabolites were identified in the following successive steps. (1) All the metabolites with DMEAN_*j*_ < 0 were removed. (2) The Mann-Whitney *U* test was used in the remaining metabolites to check the significance of the difference between the two distributions. (3) The metabolites were ranked using the *P* value. The metabolites with *P* values smaller than the predefined threshold (e.g., 0.01) were considered informative metabolites, whereas those with *P* values larger than the threshold were considered uninformative metabolites. The *P* values calculated in this manner are conditional in all other metabolites because both normal prediction errors and permuted prediction errors are dependent on all other metabolites included in all the subwindows [37, 44]. Usually, the more important a metabolite is, the higher the score assigned to it. In this case, a so-called Conditional Synergetic Score (COSS) is defined as the minus logarithm-transformed *P* value:

(1) COSSi={−log⁡10⁡(Pi),DMENi>0−log⁡10⁡(Pi+1),DMENi≤0.

Clearly, the more significant a metabolite is, the higher the score it will get. Particularly, a metabolite with *P* < 0.01 will have a COSS > 2. Thus, the informative metabolites revealed via SPA may be considered the most probable biomarker candidates.

## 3. Results

### 3.1. Clinical Characteristics of Excess and Deficiency Syndromes in Patients with DM

Clinical characteristics of the 366 subjects are summarized in Table 1. Among the 366 subjects, 308 (84.1%) were diagnosed with DM, 67 (21.8%) of which had excess syndrome. The patients with deficiency syndromes were significantly more likely to be older than those with excess syndromes in the DM group (*P* < 0.01). However, other statistical significances were not found. The systolic blood pressure, serum fasting and post-load glucose levels, and glycated hemoglobin were significantly higher in subjects with DM compared with those without DM (*P* < 0.001). However, opposite results were found for incorporative hyperlipidemia (*P* < 0.001).

### 3.2. GC/MS Profiles of Urine Samples

**Figure 1:**
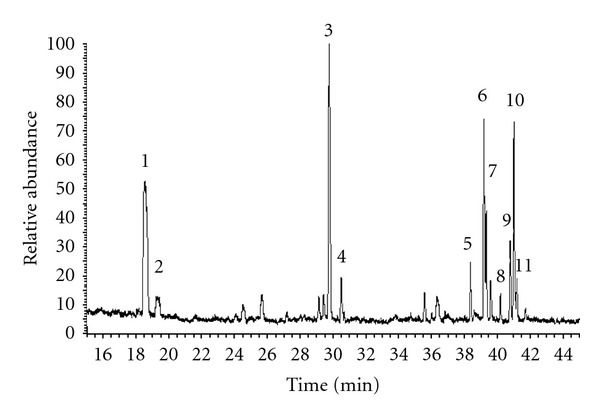
GC/MS profiles of carbohydrate metabolites from urine of the DM patients: (1) C_4_ sugar 1, (2) C_4_ sugar 2, (3) arabinose, (4) xylose, (5) inositol A, (6) allose (the internal standard), (7) inositol C, (8) talose, (9) mannose, (10) glucose, and (11) inositol D.

Based on the previously developed method and related literature [45], the GC/MS parameters were optimized for the Thermo GC/MS system used in the present study. This system allowed the detection of several peaks from the GC/MS chromatogram within 50 min of analysis cycle. The typical total-ion chromatograms from the GC/MS of urine samples from DM patients are shown in Figure 1. Ten urinary carbohydrate metabolites were identified in patients with and without DM using standards, and their peak areas were integrated for further multivariate analysis.

### 3.3. Classification of the K-OPLS Models

All the samples were used to build models. In the present study, K-OPLS was performed using the Gaussian kernel function. *σ* and Ao were optimized using 10-fold cross-validation. Accuracy of classification of cross-validation (ACCV) was calculated for each combination of *σ* and Ao. These parameters were optimized by generating models with *σ* and Ao values of 0.1 to 10 and 1 to 10, respectively.

**Figure 2:**
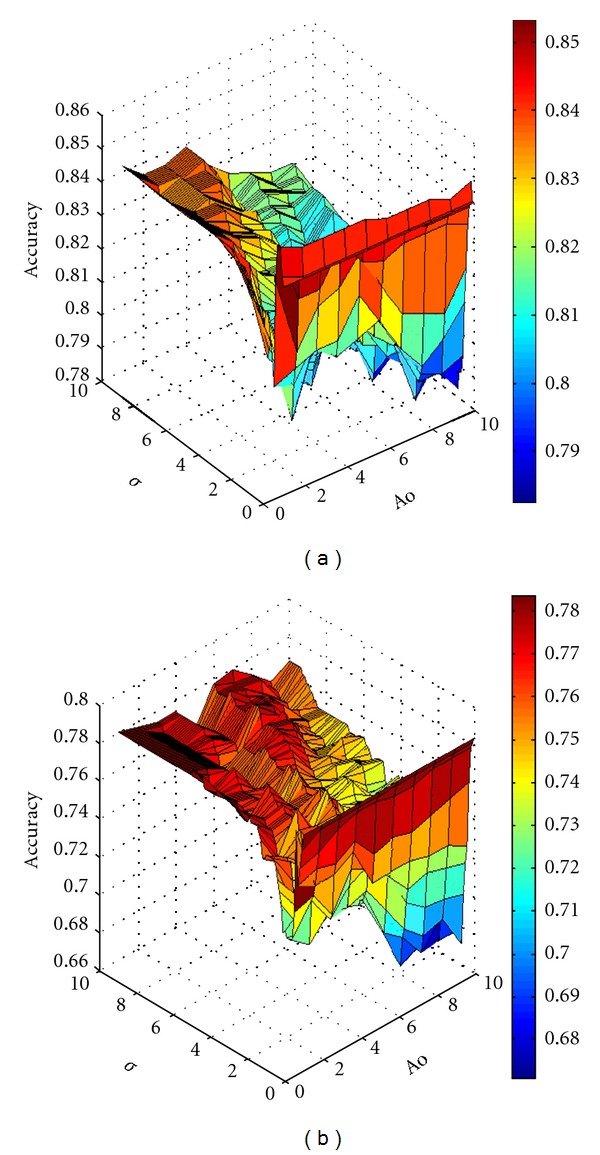
Accuracy of classification of cross-validation (ACCV) produced from each combination of *σ* and Ao parameters after cross-validation. ACCV was the largest when *σ* = 0.5 and Ao = 1 for (a) DM and non-DM subjects as well as for (b) excess and deficiency groups.

Figure 1 shows the results after cross-validation. ACCV was the largest at *σ* = 0.5 and Ao = 1 for DM and non-DM as well as for excess and deficiency syndrome groups. These optimal parameters were selected to model for these two groups, respectively (Figures 2(a) and 2(b)).

**Figure 3:**
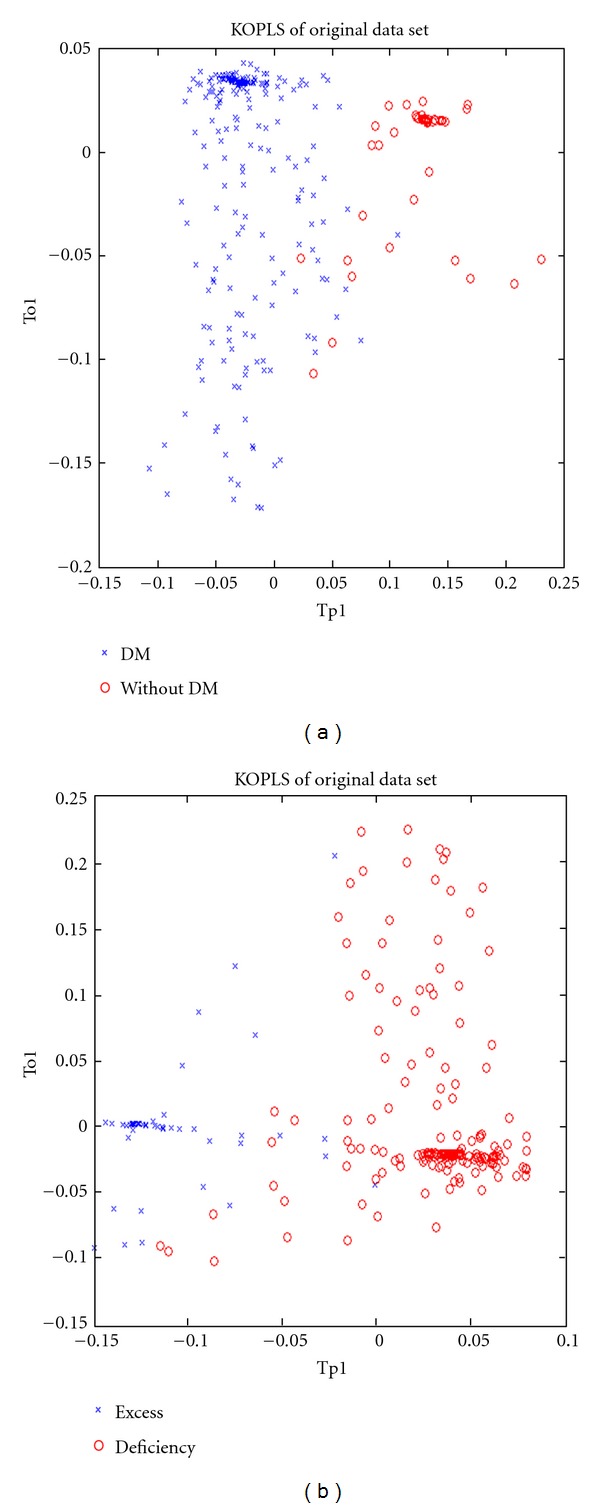
First predictive and Y-orthogonal score components, depicting how the Y-orthogonal variation was captured by the K-OPLS model. (a) Changes in the metabolic response in the urine of DM and non-DM patients. (b) Clear separation of the excess and deficiency groups.

Tenfold cross-validation was applied to evaluate the predictive abilities of the constructed K-OPLS-DA models. The primary data were divided into 10 sets. One set was the “test set,” and the others were the “training sets,” which were repeatedly calculated 10 times to obtain the components.  Table 2 shows the Q2Y, R2Y, and R2X used in evaluating all the calibration models of the two groups. R2X and R2Y were defined as the explained variation of the input (metabolic data) and output variables (disease category data), respectively. Q2Y denoted the prediction statistics over cross-validation for the classification task [46]. The values of these parameters approaching 1.0 indicate a stable model with a predictive reliability[47]. High coefficient values of R2Y and Q2Y represent good prediction [48]. As displayed by the score plots of K-OPLS (Figure 3(a)), the two sample groups can be separated into distinct clusters to indicate the changes in the metabolic response of the DM and non-DM urine samples. The samples in the excess and deficiency groups were also clearly separated (Figure 3(b)). The R2X, R2Y, and Q2Y of the former model were 0.591, 1, and 0.853, respectively, whereas those of the latter model were 0.543, 1, and 0.783, respectively (Table 2). These results indicated that the models had a good ability of explaining and predicting the variations in the X and Y matrices.

### 3.4. Differential Metabolites from SPA Based on the K-OPLS Models

For this data, the number of Monte Carlo Simulation (*N*), ratio of calibration samples to the total samples (*R*), and number of variables to be sampled in each Monte Carlo Simulation (*Q*) of SPA were set to 1000, 0.8, and 8, respectively. 

**Figure 4:**
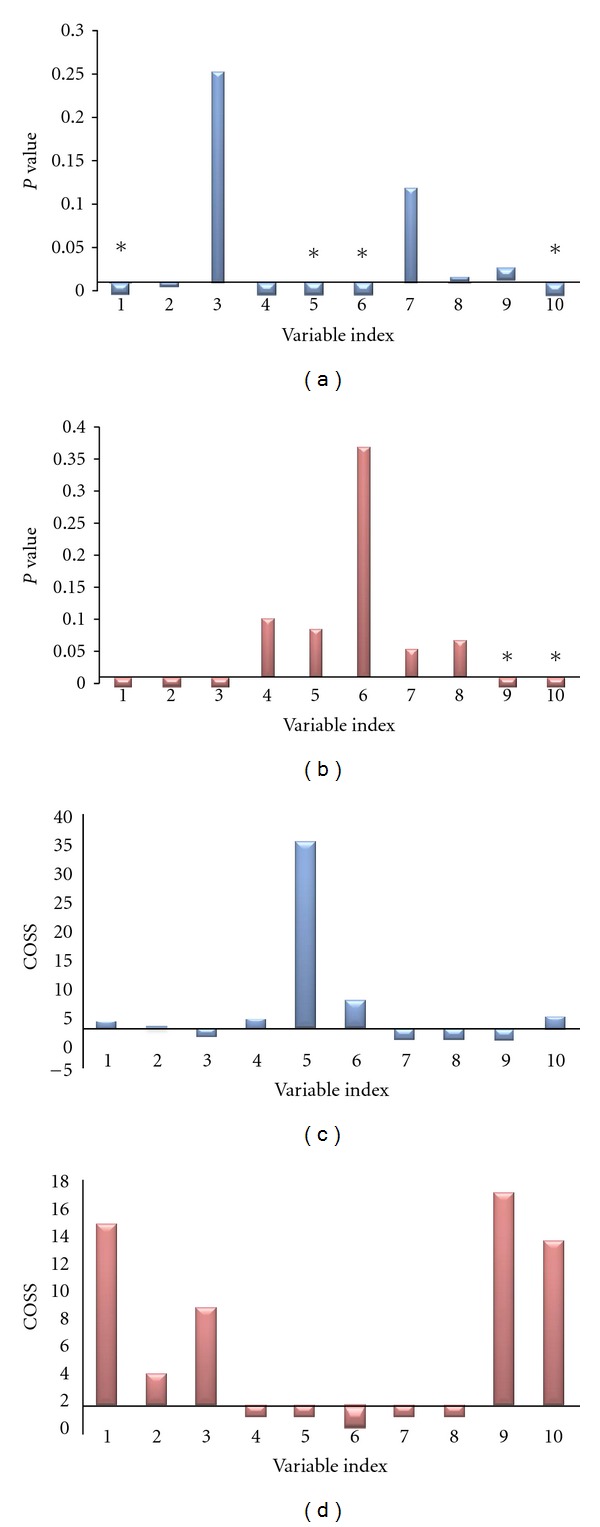
The computed *P* values and COSS through SPA for DM and non-DM group data (a and c) and excess and deficiency group data (b and d). The variable index consists of the following: (1) C_4_ sugar 1, (2) inositol C, (3) talose, (4) mannose, (5) inositol D, (6) glucose, (7) inositol A, (8) arabinose, (9) xylose, and (10) C_4_ sugar 2. *Represents *P* < 0.05 from the *t*-test between groups.

Each metabolite was first standardized with zero mean and unit variance before further analysis. With this setup, the SPA was applied to the data, and the *P* value of each metabolite was computed through the Mann-Whitney *U* test (Figures 4(a) and 4(b)). The corresponding COSS for each metabolite is shown in Figures 4(c) and 4(d). 

The two plots of DM and non-DM data obviously suggest that metabolites, including C_4_ sugar 1, inositol C, mannose, inositol D, glucose, and C_4_ sugar 2, were of small *P* values (smaller than 0.01) and COSS > 2. These six metabolites may possibly be formative metabolites or biomarkers. Thus, they should be included in further analysis. The remaining four metabolites were of high *P* values and COSS < 2. The first six significant metabolites were selected to have the best metabolite patterns, which collectively showed high prediction abilities in the clinical outcome. Combined with the *t*-test results (*P* < 0.05), the four metabolites were as follows: C_4_ sugar 1, inositol D, glucose, and C_4_ sugar 2. Similarly, the variables C_4_ sugar 1, C_4_ sugar 2, inositol C, talose, and xylose were found to have *P* < 0.01 and COSS > 2 in the excess and deficiency group data. However, based on the *t*-test results, only xylose and C_4_ sugar 2 were statistically significant in the two groups.

## 4. Discussion

TCM is a medical system with at least 3000 years of uninterrupted clinical practice. It has the advantage of collecting macroscopic information of a patient for diagnosis, with syndrome as the core of diagnosis and therapy in TCM [49]. Nowadays, the diagnosis of syndromes in TCM mainly relies on four examinations (inspection, listening and smelling examinations, inquiry, and palpation). Outcomes of TCM diagnoses may lack consistency among TCM doctors [50, 51]. Thus, the accuracy is relatively low. The use of objective indices in syndrome diagnosis in TCM may significantly improve accuracy.

Until now, syndromes in TCM have always been studied in a specific disease or biomedical condition. In addition, several studies have demonstrated that syndromes are significantly associated with diseases [49, 52, 53]. However, the biological basis of a syndrome in the context of a disease is rarely studied. The issue is significantly critical because it not only establishes a diagnostic avenue in a microcosmic level but also divides the disease into several subtypes and provides a basis for individual therapy. The establishment of a diagnostic method in the microcosmic level is an urgent and major problem in TCM [54].

DM is characterized by two major defects: a dysregulation in pancreatic hormone secretion and a decrease in insulin action on target tissues (insulin resistance). These abnormalities are related to several defects in insulin-signaling mechanisms and several steps in regulating glucose metabolism (transport and key enzymes of glycogen synthesis or mitochondrial oxidation) [55]. The development of strategies to diagnose, prevent, or delay the progression of DM has gained increasing interest because of its high morbidity and mortality rates. TCM has played an important role in lowering blood glucose and controlling the development of DM. Many studies have shown that TCM, such as Radix Astragali, Radix Rehmanniae, and Radix Trichosanthis, also has hypoglycemic effects [56]. Thus, the present study was designed to determine whether metabolomics is useful and powerful enough to differentiate between the deficiency and excess syndromes of TCM using DM as a model.

The systolic blood pressure, serum concentrations of fasting and post-load glucose, and glycated hemoglobin were significantly higher in subjects with DM than in those without DM. This result is in accordance with the characteristics of diabetes. By contrast, no clear difference was found between the two groups. This result reflects that the two subject groups had relative backgrounds in terms of age, sex, waist circumference, hip circumference, WHR, diastolic blood pressure, TG, ALT, VLDL, and HDL levels, except for the incidence of incorporative hyperlipidemia. 

The deficiency syndrome patients were older than the excess. This finding is in agreement with the TCM theory that Qi, Xue, Yin, and Yang are more insufficient in older than in younger people. However, other differences including biochemical values were not found between the two groups. This result implies that the TCM syndromes are difficult to differentiate using the clinical biochemical indicators. Therefore, TCM syndromes should be distinguished using other methods. 

Considering the intrinsic relationship between TCM theory and systems biology, some researchers began to discuss the prospective application of metabolomics to TCM theory. Metabolic profiling has been recently exploited in the pathophysiological studies of diseases [57–60]. However, only a few reports concerning the metabolomics approach in TCM research have been found in the current literature [61, 62]. In the present study, a GC/MS-based metabolomic approach was used for determining the biochemical profiles of different TCM syndrome types in DM. Moreover, the method was also used in testing whether the metabolomics approach is powerful enough to differentiate TCM syndrome types.

With the development of metabolomics, the data-mining technique has become increasingly mature. Its advantages are very applicable to the complex correlativity study of TCM syndromes and metabolites. However, the relationship between disease and metabolic data displayed nonlinear characteristics in the present study. Therefore, good models were not performed using the PLSLDA or OPLSDA method, such as R2X < 0.3 or Q2Y < 0.1. The nonlinear classification model K-OPLS had later shown stronger classification ability than the PLSLDA and OPLSDA linear classifiers. 

In the present study, we first discovered that the comprehensive differences of metabolic intermediates between subjects with and without DM focused mainly on those involved in glucose metabolism. The study identified ten carbohydrate compositions, including C_4_ sugar 1, inositol C, talose, mannose, inositol D, glucose, inositol A, arabinose, xylose, and C_4_ sugar 2. Based on the results of K-OPLS/SPA, six and five possible markers with *P* < 0.01 and COSS > 2 were found in DM and non-DM subjects and excess and deficiency groups, respectively. *T*-test was also used to compute the *P* value for each metabolite. Clearly, the results of *t*-test were not comparable with those of SPA. Two or three of them had no significant difference between groups based on the *t*-test (*P* > 0.05), further suggesting that the conditional *P* value calculated via SPA was much more informative. The main reason may be that the variable importance computed using SPA can reflect the synergetic effect to some extent [44]. Therefore, one metabolite may not be alone in a disease status but interacts with other metabolites. 

Consequently, four intermediates including inositol D, C_4_ sugar 2, glucose, and C_4_ sugar 1 produced during glycolysis were elevated in the DM group samples. The high prediction performance of the four metabolites indicates that they are possible biomarker candidates for DM. Furthermore, two potential biomarkers, xylose and C_4_ sugar 2, were discovered in the two syndromes using K-OPLS/SPA and *t*-test. These potential biomarkers can be identified by the MS database and corresponding standards.

Metabolites are endogenous and exogenous molecules that play a role in cellular regulatory and biological systems. Glucose is the major source of energy production and macromolecule biosynthesis in maintaining the normal state of the body. Highly active glycolysis and an impaired Krebs cycle guarantee enough metabolic intermediates by avoiding thorough oxidation of glucose. This phenomenon is essential for the synthesis of macromolecules, such as lipid, protein, and nuclear acid, during cell division [63–65]. The circulating glucose is filtrated by the glomerulus and absorbed by the renal tubules. Therefore, healthy human urine should not contain any sugar. Hyperglycemia, other metabolic disorders, and chronic complications due to an absolute lack of insulin and/or a reduction of the biological effects of insulin may cause the appearance of corresponding sugars in urinary metabolites. For example, 4-carbon sugars are the intermediate products of glucose metabolism. Inositol, a water-soluble vitamin, can play insulin-like roles on a metabolic enzyme. Mannose is a sugar monomer of the aldohexose series of carbohydrates and a C-2 epimer of glucose. It cannot be metabolized well in vivo. Hence, 90% of mannose will be discharged through the urine within 30 min to 60 min, and 99% of mannose in residual urine will be excreted in the next 8 h. Arabinose is a monosaccharide containing five carbon atoms and is decomposed into glucose and fructose by intestinal sucrose. Sucrose is involved in amino and nucleotide sugar metabolisms. Xylose is the connection unit between the sugar chain and serine or threonine as a combined form in vivo. Talose, also called hydrolysis of lactose, has an unknown significance so far. Therefore, the above components were present in the urine of DM patients. This finding indicates the presence of significant glucose metabolism disorders in diabetes.

Metabolic profiling can sensitively reflect all physiological and pathological changes. Moreover, it can elucidate the “syndrome” concept in TCM complex physiological systems. Using all metabolites in the evaluation of the human health status is more accurate and comprehensive than using a single index [66, 67]. The present study indicated that xylose and C_4_ sugar 2 were higher in the excess than in the deficiency group. Therefore, the holistic application of metabolic profiling in studying the syndrome essence of TCM is reasonable. In summary, these potential biomarkers reflected the deregulation of glucose metabolism in diabetic individuals, which might help in DM diagnosis and TCM syndrome differentiation.

## 5. Conclusions

This research strongly supported that metabolic profiling analysis combined with K-OPLS and SPA is a powerful tool in revealing metabolic differences between various groups, obtaining valuable information to probe molecular mechanisms, and discovering the scientific connotation of TCM theory. Larger randomized trials with an appropriate methodology, including the study of diabetic patients with different TCM syndromes, are required to confirm the results of the present study.

## Figures and Tables

**Table 1:** Clinical and biological characteristics of excess and deficiency syndromes in patients with DM.

	Subjects with DM (*n* = 308)				Subjects without DM (*n* = 58)	
	Total	Excess	Deficiency	*P* ^ a^	Total	*P* ^ b^
Gender (male/female, *n*)	308(116/192)	67(27/40)	241(89/152)	0.718	58(22/36)	0.969
Age (year)	70.32 ± 9.08	65.09 ± 9.71	71.77 ± 8.36	<0.001	67.84 ± 10.84	0.066
BMI (kg/m^2^)	25.28 ± 2.96	24.92 ± 3.01	25.38 ± 2.92	0.261	26.10 ± 3.11	0.056
Waist circumference (cm)	91.01 ± 8.42	89.88 ± 8.70	91.32 ± 8.33	0.213	91.55 ± 8.22	0.654
Hip circumference (cm)	101.24 ± 7.58	100.50 ± 7.81	101.44 ± 7.52	0.358	101.53 ± 6.33	0.781
Waist-to-hip ratio (WHR)	0.90 ± 0.06	0.89 ± 0.05	0.90 ± 0.06	0.439	0.90 ± 0.07	0.743
Obese (BMI ≥ 25)	51.3%(158/308)	49.3%(33/67)	51.9%(125/241)	0.810	60.3%(35/58)	0.262
Hypertension	93.2%(287/308)	89.6%(60/67)	94.2%(227/241)	0.290	98.3%(57/58)	0.232
Hyperlipidemia	39.3%(121/308)	37.3%(25/67)	39.8%(96/241)	0.816	81.0%(47/58)	<0.001
Coronary heart disease	21.8%(67/308)	23.9%(16/67)	21.2%(51/241)	0.757	25.9%(15/58)	0.605
Cerebrovascular accident	0.07%(22/308)	0.06%(4/67)	0.07%(8/241)	0.878	0.07%(4/58)	1.000
Hyperuricemia	0.07%(23/308)	0.06%(4/67)	0.08%(19/241)	0.791	0.09%(5/58)	0.973
Fatty liver disease	75.3%(232/308)	67.2%(45/67)	77.6%(187/241)	0.112	87.9%(51/58)	0.053
SBP (mmHg)	138.68 ± 14.37	137.34 ± 13.71	139.05 ± 14.55	0.391	133.00 ± 14.22	0.006
DBP (mmHg)	78.70 ± 9.44	79.97 ± 9.23	78.35 ± 9.48	0.209	79.10 ± 8.84	0.764
FPG (mmol/L)	7.43 ± 1.95	7.56 ± 2.21	7.39 ± 1.88	0.994	5.78 ± 1.41	<0.0001
2h PG (mmol/L)	11.25 ± 3.70	11.21 ± 3.54	11.26 ± 3.75	0.918	7.69 ± 3.30	<0.0001
HbA1c (%)	7.25 ± 1.35	7.13 ± 1.31	7.28 ± 1.36	0.461	6.17 ± 0.96	<0.0001
TG (mmol/L)	1.53 ± 0.92	1.45 ± 0.79	1.56 ± 0.96	0.908	1.87 ± 1.31	0.068
HDL cholesterol (mmol/L)	1.34 ± 0.36	1.31 ± 0.26	1.35 ± 0.39	0.473	1.39 ± 0.39	0.363
AST (U/L)	25.21 ± 12.83	27.14 ± 13.49	24.68 ± 12.61	0.171	26.38 ± 13.93	0.537
VLDL cholesterol (mmol/L)	2.56 ± 0.56	2.55 ± 0.59	2.56 ± 0.55	0.951	2.48 ± 0.57	0.322

^a^ *P* value refers to the comparison between excess versus deficiency syndromes within the DM group. ^b^ *P* value refers to the comparison between subjects with and without DM using chi-square test or *t*-test analysis.

**Table 2:** Results of prediction of the K-OPLS models.

Models	*σ*	Ao	R2X	R2Y	Q2Y
DM and non-DM	0.5	1	0.591	1.000	0.853
Excess and deficiency	0.5	1	0.543	1.000	0.783

## References

[1] Wild S, Roglic G, Green A, Sicree R, King H (2004). Global prevalence of diabetes: estimates for the year 2000 and projections for 2030. *Diabetes Care*.

[2] Yi L, He J, Liang Y, Yuan D, Gao H, Zhou H (2007). Simultaneously quantitative measurement of comprehensive profiles of esterified and non-esterified fatty acid in plasma of type 2 diabetic patients. *Chemistry and Physics of Lipids*.

[3] Castell C (2010). Epidemiology and classification for diabetes mellitus. *Revista de Enfermería*.

[4] Ning G, Hong J, Bi Y (2009). Progress in diabetes research in China. *Journal of Diabetes*.

[5] Wang LM, Zhao X, Wu XL (2012). Diagnosis analysis of 4 TCM patterns in sub-optimal health status: a structural equation modeling approach. *Evidence-Based Complementary and Alternative Medicine*.

[6] Nicholson JK, Connelly J, Lindon JC, Holmes E (2002). Metabonomics: a platform for studying drug toxicity and gene function. *Nature Reviews Drug Discovery*.

[7] Malandrino N, Smith RJ (2011). Personalized medicine in diabetes. *Clinical Chemistry*.

[8] Griffin JL, Vidal-Puig A (2008). Current challenges in metabolomics for diabetes research: a vital functional genomic tool or just a ploy for gaining funding?. *Physiological Genomics*.

[9] van Doorn M, Vogels J, Tas A (2007). Evaluation of metabolite profiles as biomarkers for the pharmacological effects of thiazolidinediones in type 2 diabetes mellitus patients and healthy volunteers. *British Journal of Clinical Pharmacology*.

[10] Shaham O, Wei R, Wang TJ (2008). Metabolic profiling of the human response to a glucose challenge reveals distinct axes of insulin sensitivity. *Molecular Systems Biology*.

[11] Wopereis S, Rubingh CM, van Erk MJ (2009). Metabolic profiling of the response to an oral glucose tolerance test detects subtle metabolic changes. *PLoS ONE*.

[12] Zhao X, Peter A, Fritsche J (2009). Changes of the plasma metabolome during an oral glucose tolerance test: is there more than glucose to look at?. *American Journal of Physiology - Endocrinology and Metabolism*.

[13] Chen J, Zhao X, Fritsche J (2008). Practical approach for the identification and isomer elucidation of biomarkers detected in a metabonomic study for the discovery of individuals at risk for diabetes by integrating the chromatographic and mass spectrometric information. *Analytical Chemistry*.

[14] Plumb RS, Johnson KA, Rainville P (2006). The detection of phenotypic differences in the metabolic plasma profile of three strains of Zucker rats at 20 weeks of age using ultra-performance liquid chromatography/orthogonal acceleration time-of-flight mass spectrometry. *Rapid Communications in Mass Spectrometry*.

[15] Shearer J, Duggan G, Weljie A, Hittel DS, Wasserman DH, Vogel HJ (2008). Metabolomic profiling of dietary-induced insulin resistance in the high fat-fed C57BL/6J mouse. *Diabetes, Obesity and Metabolism*.

[16] Toye AA, Dumas ME, Blancher C (2007). Subtle metabolic and liver gene transcriptional changes underlie diet-induced fatty liver susceptibility in insulin-resistant mice. *Diabetologia*.

[17] Williams RE, Lenz EM, Rantalainen M, Wilson ID (2006). The comparative metabonomics of age-related changes in the urinary composition of male Wistar-derived and Zucker (fa/fa) obese rats. *Molecular BioSystems*.

[18] Williams R, Lenz EM, Wilson AJ (2006). A multi-analytical platform approach to the metabonomic analysis of plasma from normal and Zucker (fa/fa) obese rats. *Molecular BioSystems*.

[19] Mäkinen VP, Soininen P, Forsblom C (2008). 1H NMR metabonomics approach to the disease continuum of diabetic complications and premature death. *Molecular Systems Biology*.

[20] Zhang S, Nagana Gowda GA, Asiago V, Shanaiah N, Barbas C, Raftery D (2008). Correlative and quantitative 1H NMR-based metabolomics reveals specific metabolic pathway disturbances in diabetic rats. *Analytical Biochemistry*.

[21] Gipson GT, Tatsuoka KS, Ball RJ (2008). Multi-platform investigation of the metabolome in a leptin receptor defective murine model of type 2 diabetes. *Molecular BioSystems*.

[22] Huo T, Cai S, Lu X, Sha Y, Yu M, Li F (2009). Metabonomic study of biochemical changes in the serum of type 2 diabetes mellitus patients after the treatment of metformin hydrochloride. *Journal of Pharmaceutical and Biomedical Analysis*.

[23] Salek RM, Maguire ML, Bentley E (2007). A metabolomic comparison of urinary changes in type 2 diabetes in mouse, rat, and human. *Physiological Genomics*.

[24] van Doorn M, Vogels J, Tas A (2007). Evaluation of metabolite profiles as biomarkers for the pharmacological effects of thiazolidinediones in type 2 diabetes mellitus patients and healthy volunteers. *British Journal of Clinical Pharmacology*.

[25] Zhang X, Wang Y, Hao F (2009). Human serum metabonomic analysis reveals progression axes for glucose intolerance and insulin resistance statuses. *Journal of Proteome Research*.

[26] Bao Y, Zhao T, Wang X (2009). Metabonomic variations in the drug-treated type 2 diabetes mellitus patients and healthy volunteers. *Journal of Proteome Research*.

[27] Li H, Ni Y, Su M (2007). Pharmacometabonomic phenotyping reveals different responses to xenobiotic intervention in rats. *Journal of Proteome Research*.

[28] Bain JR, Stevens RD, Wenner BR, Ilkayeva O, Muoio DM, Newgard CB (2009). Metabolomics applied to diabetes research: moving from information to knowledge. *Diabetes*.

[29] Wang C, Kong H, Guan Y (2005). Plasma phospholipid metabolic profiling and biomarkers of type 2 diabetes mellitus based on high-performance liquid chromatography/electrospray mass spectrometry and multivariate statistical analysis. *Analytical Chemistry*.

[30] Yi LZ, He J, Liang YZ, Yuan DL, Chau FT (2006). Plasma fatty acid metabolic profiling and biomarkers of type 2 diabetes mellitus based on GC/MS and PLS-LDA. *FEBS Letters*.

[31] Williams R, Lenz EM, Wilson AJ (2006). A multi-analytical platform approach to the metabonomic analysis of plasma from normal and Zucker (fa/fa) obese rats. *Molecular BioSystems*.

[32] Goodacre R, Broadhurst D, Smilde AK (2007). Proposed minimum reporting standards for data analysis in metabolomics. *Metabolomics*.

[33] Lin X, Wang Q, Yin P (2011). A method for handling metabonomics data from liquid chromatography/mass spectrometry: combinational use of support vector machine recursive feature elimination, genetic algorithm and random forest for feature selection. *Metabolomics*.

[34] Rantalainen M, Bylesjö M, Cloarec O, Nicholson JK, Holmes E, Trygg J (2007). Kernel-based orthogonal projections to latent structures (K-OPLS). *Journal of Chemometrics*.

[35] Bylesjö M, Rantalainen M, Nicholson JK, Holmes E, Trygg J (2008). K-OPLS package: kernel-based orthogonal projections to latent structures for prediction and interpretation in feature space. *BMC Bioinformatics*.

[36] Cao DS, Liang YZ, Xu QS, Li HD, Chen X (2010). A new strategy of outlier detection for QSAR/QSPR. *Journal of Computational Chemistry*.

[37] Li HD, Liang YZ, Xu QS, Cao DS (2010). Model population analysis for variable selection. *Journal of Chemometrics*.

[38] Li X, Xu Z, Lu X (2009). Comprehensive two-dimensional gas chromatography/time-of-flight mass spectrometry for metabonomics: biomarker discovery for diabetes mellitus. *Analytica Chimica Acta*.

[39] World Health Organisation (1999). Definition, diagnosis, and classification of diabetes mellitus and its complications. *Report of a WHO Consultation. Part 1: Diagnosis and Classification of Diabetes Mellitus*.

[40] Zheng YY (2002). *Guiding Principle of Clinical Research on New Drugs of Traditional Chinese Medicine*.

[41] Choo V (2002). WHO reassesses appropriate body-mass index for Asian populations. *The Lancet*.

[42] Yang M, Chen JL, Shi XF, Niu HJ (2011). Rapid determination of aesculin, aesculetin and fraxetin in cortex fraxini extract solutions based on ultraviolet spectroscopy. *E-Journal of Chemistry*.

[43] Filzmoser P, Liebmann B, Varmuza K (2009). Repeated double cross validation. *Journal of Chemometrics*.

[44] Li HD, Zeng MM, Tan BB, Liang YZ, Xu QS, Cao DS (2010). Recipe for revealing informative metabolites based on model population analysis. *Metabolomics*.

[45] Xie JH, Xie MY, Nie SP, Shen MY, Wang YX, Li C (2010). Isolation, chemical composition and antioxidant activities of a water-soluble polysaccharide from *Cyclocarya paliurus* (Batal.) Iljinskaja. *Food Chemistry*.

[46] Yuan K, Kong H, Guan Y, Yang J, Xu G (2007). A GC-based metabonomics investigation of type 2 diabetes by organic acids metabolic profile. *Journal of Chromatography B*.

[47] Ni Y, Wang Y, Kokot S (2008). Multicomponent kinetic spectrophotometric determination of pefloxacin and norfloxacin in pharmaceutical preparations and human plasma samples with the aid of chemometrics. *Spectrochimica Acta A*.

[48] Girard J (2005). Contribution of free fatty acids to impairment of insulin secretion and action. Mechanism of *β*-cell lipotoxicity. *Medecine/Sciences*.

[49] Zhao HH, Chen JX, Hou N (2011). Discovery of diagnosis pattern of coronary heart disease with Qi Deficiency syndrome by the T-test-based adaboost algorithm. *Evidence-Based Complementary and Alternative Medicine*.

[50] Sung JJY, Leung WK, Ching JYL (2004). Agreements among traditional Chinese medicine practitioners in the diagnosis and treatment of irritable bowel syndrome. *Alimentary Pharmacology and Therapeutics*.

[51] Zhang GG, Bausell B, Lao L, Lee WL, Handwerger B, Berman B (2004). The variability of TCM pattern diagnosis and herbal prescription on rheumatoid arthritis patients. *Alternative Therapies in Health and Medicine*.

[52] Li S, Zhang ZQ, Wu LJ, Zhang XG, Li YD, Wang YY (2007). Understanding ZHENG in traditional Chinese medicine in the context of neuro-endocrine-immune network. *IET Systems Biology*.

[53] Kang GL, Li S, Zhang JF (2008). Entropy-based model for interpreting life systems in traditional Chinese medicine. *Evidence-based Complementary and Alternative Medicine*.

[54] Chiu PH, Hsieh HY, Wang SC (2012). Prescriptions of traditional chinese medicine are specific to cancer types and adjustable to temperature changes. *PLoS One*.

[55] Trygg J, Wold S (2002). Orthogonal projections to latent structures (O-PLS). *Journal of Chemometrics*.

[56] Xie W, Du L (2011). Diabetes is an inflammatory disease: evidence from traditional Chinese medicines. *Diabetes, Obesity and Metabolism*.

[57] Coen M, Holmes E, Lindon JC, Nicholson JK (2008). NMR-based metabolic profiling and metabonomic approaches to problems in molecular toxicology. *Chemical Research in Toxicology*.

[58] Lindon JC, Holmes E, Bollard ME, Stanley EG, Nicholson JK (2004). Metabonomics technologies and their applications in physiological monitoring, drug safety assessment and disease diagnosis. *Biomarkers*.

[59] Nicholson JK, Lindon JC (2008). Systems biology: metabonomics. *Nature*.

[60] Schmid H, Henger A, Kretzler M (2006). Molecular approaches to chronic kidney disease. *Current Opinion in Nephrology and Hypertension*.

[61] Qiu Y, Chen M, Su M (2008). Metabolic profiling reveals therapeutic effects of *Herba Cistanches* in an animal model of hydrocortisone-induced “kidney-deficiency syndrome”. *Chinese Medicine*.

[62] Chen M, Zhao L, Jia W (2005). Metabonomic study on the biochemical profiles of a hydrocortisone-induced animal model. *Journal of Proteome Research*.

[63] Deberardinis RJ, Sayed N, Ditsworth D, Thompson CB (2008). Brick by brick: metabolism and tumor cell growth. *Current Opinion in Genetics and Development*.

[64] Tong X, Zhao F, Thompson CB (2009). The molecular determinants of de novo nucleotide biosynthesis in cancer cells. *Current Opinion in Genetics and Development*.

[65] Hervouet E, Simonnet H, Godinot C (2007). Mitochondria and reactive oxygen species in renal cancer. *Biochimie*.

[66] Zhao LD, Wo XD, Li Y (2007). Effects of warming and tonifying kidney, yang drugs on liver mitochondria proteome of rats with kidney yang deficiency. *Chinese Journal of TCM and Pharmacy*.

[67] Goodacre R (2004). Metabolic profiling: pathways in discovery. *Drug Discovery Today*.

